# Can Force Feedback and Science Learning Enhance the Effectiveness of Neuro-Rehabilitation? An Experimental Study on Using a Low-Cost 3D Joystick and a Virtual Visit to a Zoo

**DOI:** 10.1371/journal.pone.0083945

**Published:** 2013-12-13

**Authors:** Paolo Cappa, Andrea Clerico, Oded Nov, Maurizio Porfiri

**Affiliations:** 1 Department of Mechanical and Aerospace Engineering, Sapienza University of Rome, Rome, Italy; 2 Department of Mechanical and Aerospace Engineering, Polytechnic Institute of New York University, Brooklyn, New York, United States of America; 3 Movement Analysis and Robotics Laboratory (MARLab), Neurorehabilitation Division, IRCCS Children’s Hospital “Bambino Gesù,” Roma, Italy; 4 Department of Technology Management and Innovation, Polytechnic Institute of New York University, Brooklyn, New York, United States of America; Oslo University Hospital, Norway

## Abstract

In this paper, we demonstrate that healthy adults respond differentially to the administration of force feedback and the presentation of scientific content in a virtual environment, where they interact with a low-cost haptic device. Subjects are tasked with controlling the movement of a cursor on a predefined trajectory that is superimposed on a map of New York City’s Bronx Zoo. The system is characterized in terms of a suite of objective indices quantifying the subjects’ dexterity in planning and generating the multijoint visuomotor tasks. We find that force feedback regulates the smoothness, accuracy, and duration of the subject’s movement, whereby converging or diverging force fields influence the range of variations of the hand speed. Finally, our findings provide preliminary evidence that using educational content increases subjects’ satisfaction. Improving the level of interest through the inclusion of learning elements can increase the time spent performing rehabilitation tasks and promote learning in a new context.

## Introduction

Robotic machines were identified as an effective tool in rehabilitation during the 1990s [1]. In this context, robot-mediated therapy (RMT) for upper limb rehabilitation has emerged as a viable approach [2-5]. The first study, authored by Dijkers and colleagues [6] and based on the clinical use of an industrial robot, has helped identifying critical factors, such as safety, acceptance by patients, and perceived utility for therapists. Hogan et al. [7] proposed the first ad-hoc robot, MIT-Manus, a device with two degrees of freedom that enables unrestricted movement of the shoulder and elbow joints in the horizontal plane. The commercial version of MIT-Manus, the most employed therapeutic robot for upper extremities [8], was originally conceived for adult stroke victims, and recently its use was extended to children with cerebral palsy [9,10].

These mechanical systems can be equipped with an array of sensors to record data, such as position, velocity, and joint torques, and host actuators for moving the patient’s limb in accordance to selected control strategies. With minimal therapist time commitment, robots can: (i) automatically facilitate a variety of movements, in terms of force and displacement fields, depending on the patient’s conditions and (ii) produce objective indices to estimate motor changes of patients during therapy [8,11]. Thus, the quantity (duration and frequency) and quality (task-specificity) of the interventions afforded by RMT are currently aiding neurologists and rehabilitation therapists to address the challenges faced by neuro-rehabilitation in the treatment of the upper limb [12,13]. 

Robots can be broadly classified as: (i) passive, where the system constrains the patient’s arm to a determined range of motion (without actuation); (ii) active, where the system moves the patient’s arm along a predefined path (through electromechanical actuation, pneumatics, etc.); and (iii) interactive, where the system reacts to the patient’s inputs to provide an optimal assistive strategy [14]. Moreover, these devices take the form of either an actuated robotic arm, that is, the end-effector type, or an actuated robotic suite that encloses the affected limb, that is, the exoskeletal type. 

Using end-effector devices, the patient holds a manipulandum, which experiences the robot-imposed forces. All the forces and measurements are executed at a single interface, which has the advantage of easy set-up for patients of different body sizes. Examples of current upper limb systems of the end-effector type are: MIT/IMT-Manus [11], MIME (Mirror Image Motion Enabler) [15], GENTLE/s [16,17], and REO-GO [18]. Systematic technical reviews, meta-analyses, comparison of different physiotherapy schools, effects of intensity of training, and efficacy of specific upper limb rehabilitation techniques are available in the literature [19-23]. Notably, the neural pathway associated with visual processing of movement stimuli used in upper limb RMT has been identified in a recent study based on visual functional magnetic resonance imaging (fMRI) [24] .

Despite the proved effectiveness of RMTs, their use is not a routine part of treatment in most therapeutic settings. The reason is that robots used for such analyses are often complicated to operate and costly to purchase and, therefore, not suitable for ordinary clinic or home use. Thus, considerable effort has been devoted to the development of less expensive systems that can be easily used for outpatient rehabilitation, without the supervision by a therapist. In addition, the need of providing rehabilitation treatments beyond the hospital stay has generated substantial interest in models exploring robotics-based technology to extend rehabilitation therapy and assessment to home environments [25-31]. 

Consequently, low-cost haptic devices, originally developed for gaming, hold promise in aiding RMT, whereby they can stimulate the kinaestetic senses of the user by delivering forces through the end-effector and create the illusion of contact with a rigid surface. A critical comparison of commercially available devices indicate that the Novint Falcon allows for the largest force amplitude [32,33]. In addition, the Falcon is characterized by a very low friction and a robust actuation [28] mechanism. These advantages, along with the transparency of the control interface, have enabled a wide spectrum of medical applications for the Falcon since its first usability test in [26], including: non-visual web interactions [31], finger-tip force measurement [27], and three-dimensional (3D) force stimulation [30]. In addition, recent efforts are starting to explore its use in neuro-rehabilitation treatments [34].

Beyond the medical domain, the Falcon has been shown to enrich learning experience by increasing participants’ motivation when used in educational games for normally developed children [25] and children with visual impairments [29]. In this context, several studies have demonstrated that patients’ engagement is a critical factor for the success of the rehabilitation training, whereby functional recovery is enhanced when the patients engage in the task and the rehabilitative movements become subconscious [35-38]. This calls for further exploration of RMT systems toward increasing patients’ engagement, and as a result, performance.

In this study, we demonstrate the feasibility of using the Falcon haptic joystick in a 2D virtual reality environment to administer different therapeutic treatments to healthy young adults. Specifically, we consider four different experimental conditions that are designed to dissect the roles of force feedback from the end-effector and science learning during task execution on the subjects’ response. Subjects’ response is studied through salient performance indices determined from the time trace of the end-effector and survey instruments administered after task completion. From a theoretical point of view, this work seeks to test the following hypotheses: (i) force feedback regulates the smoothness, accuracy, and duration of the subject’s movement and (ii) inclusion of science learning in the exercise increases participants’ interest in the tasks. From a methodological point of view, this study contributes to the development of a low-cost platform based on a haptic joystick to: (i) offer real-time feedback to participants as a function of end-effector motion in a 2D virtual environment; (ii) enable the administration of scientific content pertinent to the 2D virtual environment; (iii) establish a toolbox of sensory-motor performance indices to assess force feedback; and (iv) establish content-specific survey instruments to evaluate subjects’ engagement and learning. 

## Materials and Methods

### System

The system was composed of: (i) a hardware component, based on an off-the-shelf low-cost platform (Novint Falcon, Washington, PA) and (ii) a software component, developed in-house using MATLAB (Mathworks, Natick, MA) and “The Haptik Library” [39] by the University of Siena, Italy (www.haptiklibrary.org/).

**Figure 1:**
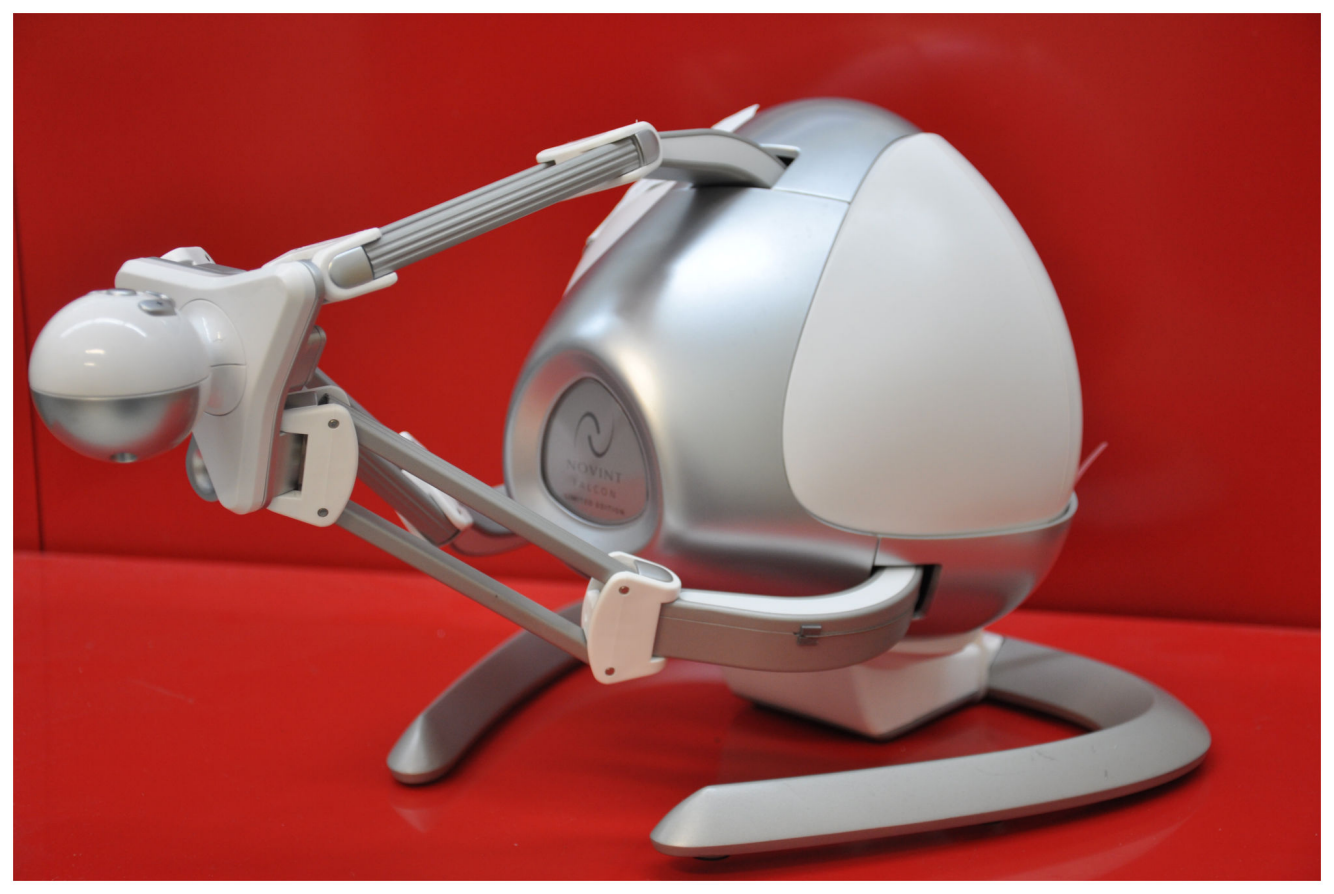
Picture of the Novint Falcon.

The Novint Falcon (Figure 1) is a relatively inexpensive haptic device developed in the gaming industry and was selected to offer force feedback while allowing the control of the end-effector with minimal effort. The end-effector of the Falcon has a workspace of 101.6 mm × 101.6 mm × 101.6 mm (4’’ × 4’’ × 4’’) and a force capability of 8.8 N (2 lbs), with controllable amplitude and direction. Although the reachable range of motion and the produced force are limited, the selected end-effector system was expected to be useful in exploring science learning in a low-cost platform. Furthermore, the use of commercially acceptable forces implicitly avoids issues of safety that exist in robotic systems developed in-house. The device collects the spatio-temporal variable of the end-effector trajectory with a frequency of 25 Hz and it applies up 1.7 N in each direction. 

**Figure 2:**
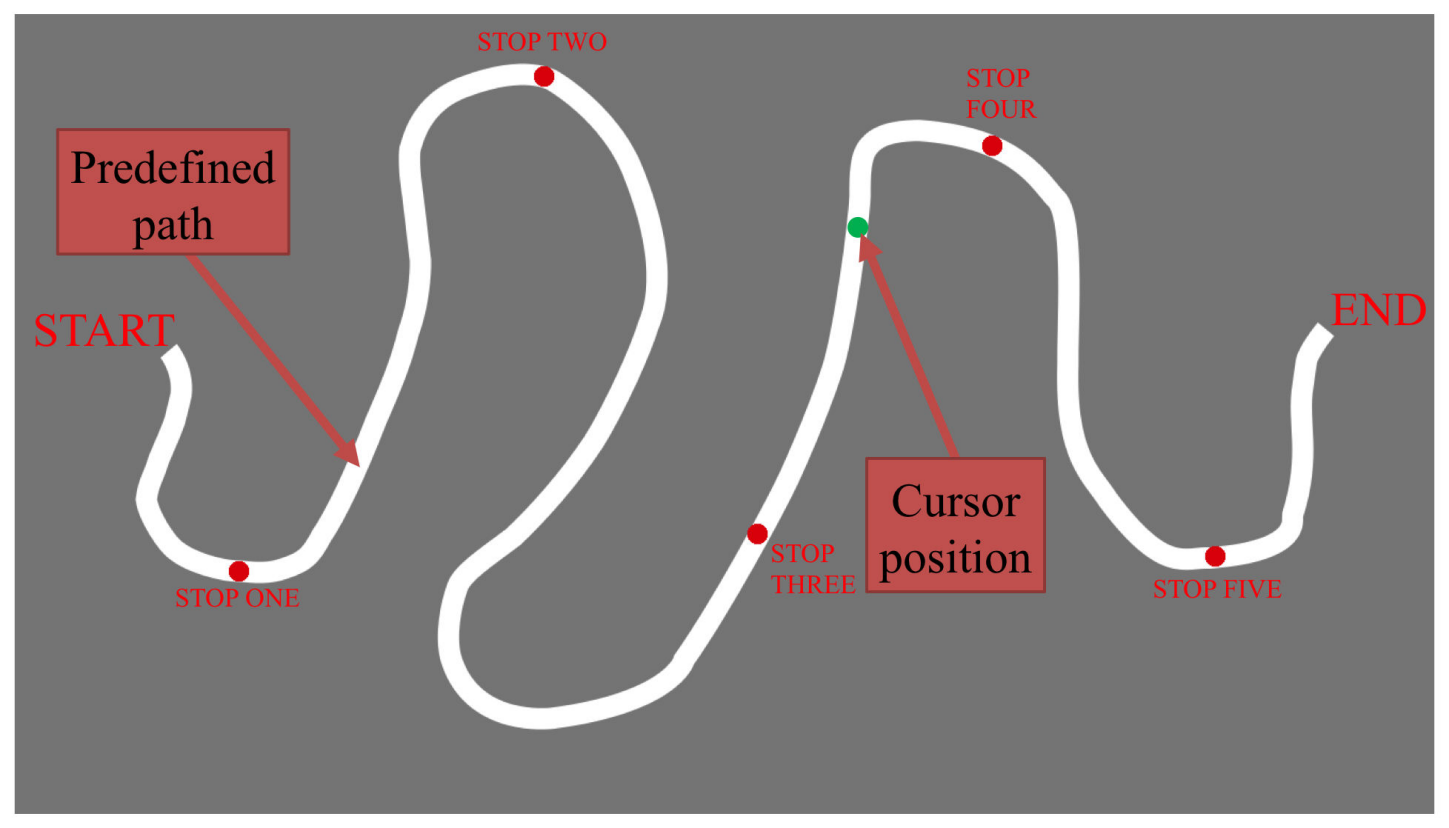
Path used for virtual feedback in our experiments displaying the moving cursor, the start and end points, and the stations where scientific content is administered. (The image presented to the subjects was superimposed on a map of the zoo and included various animal species.).

The ad-hoc 2D scenario was the representation of New York City’s Bronx Zoo (www.bronxzoo.com), where a predefined path (Figure 2) was proposed to the participants. The selection of a 2D scenario was motivated by future use of the system with clinical population with reduced visuo-spatial abilities. In each task, the position of the end-effector of the Falcon, which provided a force feedback depending on the cursor position in the map, was superimposed to the map ([0:1920] × [​​0:1080] pixels) to maintain a high level of subject’s attention throughout the trials. The criterion for reaching the target was a positioning error less than 5 mm (0.016’’) [9]; when the criterion was met, the position was indicated with a green spot, while a red spot was used to indicate a larger error. 

The system provided four different end-point behaviors: no-force feedback exerted by the system with (0F_S) or without (0F) the delivery of scientific content about the zoo; converging force (CF); and diverging force (DF). The force feedback was generated through a virtual force field, in which the predefined path corresponded to the equilibrium. The system determined the current position and velocity of the end-effector. Thus, when the cursor was on the path, no force was exerted by the manipulator and, when the cursor was not on the path, a force feedback was exerted. Such force was directed towards or away from the path depending on whether CF or DF conditions were implemented, respectively. The components of the force in the horizontal and vertical directions on the screen were obtained by performing the gradient of a potential field, which was obtained through Gaussian filtering of a separately drawn image. On average, the force feedback was on the order of 10 mN per pixel. For both conditions, a viscous damping force linearly proportional to the cursor speed, computed using a first order finite difference scheme, was utilized to smoothen the trajectories. While the Novint Falcon allows for motion and force feedback in 3D, the normal component of the force with respect to the 2D environment used to navigate the cursor is zero. Therefore, not only motion in the normal direction did not result in cursor motion, but also it did not produce force feedback in any condition and was not considered in any performance metric.

Here, we succinctly describe each of these tasks that formed the four experimental conditions of our study:

**•  No-force feedback condition (0F)**. The scenario allowed the user to move the end-effector freely within the screen and the joystick was set to exert a null force feedback. This unassisted task was designed to capture baseline trajectories to properly assess the effects induced by a force feedback (both diverging and converging) or by the scientific task.
**•  Converging field condition (CF)**. The Falcon force feedback was used to drive the participant’s hand toward the target path, thus reducing the error in the position.
**•  Diverging field condition (DF)**. The diverging field mode, in contrast with the CF mode, was used to amplify the error in the position.
**•  No-force feedback with scientific content condition (0F_S)**. In order to investigate whether a learning component can benefit performance, a fourth operating mode was implemented, wherein no force feedback was exerted, but the user was presented with content about animal species and the Bronx Zoo history along the target path. More specifically, five stops along the path were proposed to participants, each containing a paragraph of zoo-related content. Stops were approximately uniformly distributed with respect to the horizontal axis and located in correspondence of either turns or straight paths. Scientific content (adapted from www.bronxzoo.com) was presented in the form of a pop-up text window superimposed to the map of the zoo. Each window was present for approximately fifteen seconds (sixteen to twenty seconds depending on the length of text), and the cursor was automatically held in place during the reading and until the subjects were to resume the path following. For example, the following information was presented to the participants: “Fordham University owned the land which become the Bronx Zoo. Fordham sold it to the City of New York for only $1000 under the condition that the lands be used for a zoo”; “Ota Benga, a Congolese Mbuti Pygmy, in 1906 was exhibited in the Bronx Zoo’s Monkey House as an example of “earlier stages” of human evolution. In the early 20th century racial theories were frequently intertwined with concept from evolutionary biology”; and “Seal or sea lion? To tell the difference, look at the ears. Sea lions have tiny flaps over their ears and seals have none”.

### Participants

The study was carried out on a sample of 48 healthy subjects (27 males and 21 female; age 26.4 ± 3.1). The inclusion criteria were: 20 to 35 years of age; no current or previous motor and neurological disorders; no medical condition that could affect their upper limb movement performance or vision of the video screen; and, finally, no current or previous experience with the Falcon haptic interface. All participants were uninformed about the purpose of the study. 

Personal information questions addressed the participants’ demographics and their dominant hand. Written consent was obtained from all the subjects and the study was approved by the Institution Review Board at the Polytechnic Institute of New York University.

### Protocol

Participants were made to sit comfortably on a chair in front of a table, whereupon both the Falcon and the video screen were placed. Subjects were instructed to sit with the center of the video screen in the sagittal plane, and no constraint was imposed on their posture and on the pose of their arms. The only instructions provided by the experimenter to the subjects were to use their dominant hand, follow the track with minimal deviations from it, and read zoo-related content when present. No time restriction was provided on the duration of task completion. Thus, we tested the ability of healthy adults to adapt upper limb movements to spatial constraints based on incoming visual feedback, in 0F and 0F_S, and visual and force feedback, in CF and DF. Each participant conducted the four tasks. The four sets of experiments were fully randomized in their presentation to the participants, so that each possible sequence of tasks (numbering 24 possible sequences in total) was administered to two participants.

After completing the four tasks, each subject was administered a 21 item survey, with questions assessing learning from zoo-related content presented to them 0F_S as well as satisfaction from each task and personal information. Science learning was measured as the number of correct responses to ten multiple-choice informative questions about the content covered during the experiment. Questions included: “Why do biomedical researchers study toxins from frogs?” and “Which species has tiny flaps over their ears?” Satisfaction was measured on a five point Likert scale ranging between “very dissatisfied” and “very satisfied” [40]. 

### Indices

Studies on sensory-motor performance have identified a multitude of indices to quantify smoothness and coordination for investigating the effect of age, disease, or therapeutic intervention. Among feasible measures proposed in the literature on neuro-rehabilitation of the upper limb [41,42] to characterize movement smoothness, movement accuracy, and tracking rapidity, we have selected the following indices: (i) the speed metric (SM), calculated as the mean of the speed divided by the peak speed (between zero and one); (ii) the number of submovements (NSM), obtained by segmenting the movement on the basis of local maxima of the speed exceeding a set threshold value [43,44]; (iii) the deviation (D), defined as the ratio of the area between the actual and the target path and the actual path length (expressed in pixels) [45]; (iv) the normalized path length (NPL), defined as the ratio of the actual and the desired path [46]; and (v) the time duration of the trial (T) (expressed in seconds). SM and NSM were selected to measure the movement smoothness (low values of SM and NSM indicate smooth movements) [47]; D and NPL were used to quantify the movement accuracy (low values of D indicate accurate movements and low values of NPL identify a nearly rectilinear trajectory from the start to the end points); and T was utilized to measure the tracking speed [46] (low values of T represent rapidly executed tasks).

The onset and the termination of a movement were detected by processing the hand speed off-line and a threshold value of 0.05 m/s (1.97 in/s) was used to partition the individual trajectory of a participant in submovements [48]. The indices were computed over a time window starting with the cursor departing from the start point and ending when reaching the final target. For 0F_S, the time in which zoo-related content was presented to the participants was excluded from the analysis. 

### Statistical Analysis

To ascertain differences in the sensory-motor performance for each index among the participants, a one-way analysis of variance (ANOVA) was used. Specifically, for each of the five indices (SM, NSM, D, NPL, and T), we compared the mean response under the four experimental conditions (0F, CF, DF, and 0F_S). Based on the significant main effect of condition, Fisher’s protected least significant difference (PLSD) post-hoc tests were used. 

To assess the impact of the presentation of scientific content on participants’ satisfaction, a paired-samples t-test was performed comparing the learning-based task and the control task, that is, 0F_S and 0F. 

To test the hypothesis that learning is positively associated with satisfaction from the task, the following procedure was carried out: for each participant, the difference between satisfaction in 0F_S and 0F was calculated. For subjects who indicated a difference in such satisfaction, a Pearson correlation analysis was carried out between the satisfaction difference level and the number of correct answers in the content test. 

Statistical analyses were performed with built-in functions of Statview 5.0 (Abacus, Berkeley, CA) and SPSS 20 (IBM, Armonk, NY). The significance level was set at p < 0.05 for all statistical tests.

## Results

### Force feedback and scientific learning regulate movement smoothness (Figure 3)

Our results support the expectation that the smoothness of participants’ movements is influenced by the experimental condition (for SM F3,48=30.256, p<0.0001 and for NSM F3,48=13.072, p<0.0001). Specifically, the speed metric SM is maximized for the control condition 0F in which participants were not subject to either force feedback or scientific learning (p<0.0001 in post-hoc tests comparing 0F to any other condition). Moreover, the presence of a converging feedback in CF increases the speed metric as compared to the conditions in which diverging feedback or scientific learning are present, that is, DF or 0F_S (p<0.0005 in post-hoc tests). In addition, an analysis of the number of submovements NSM indicates that the force feedback contributed to reducing the partitioning of the subject’s tracks in the trials (p<0.0001 in post-hoc tests comparing CF or DF to either 0F or 0F_S). No significant differences in NSM are observed when comparing 0F with 0F_S and CF with DF.

**Figure 3:**
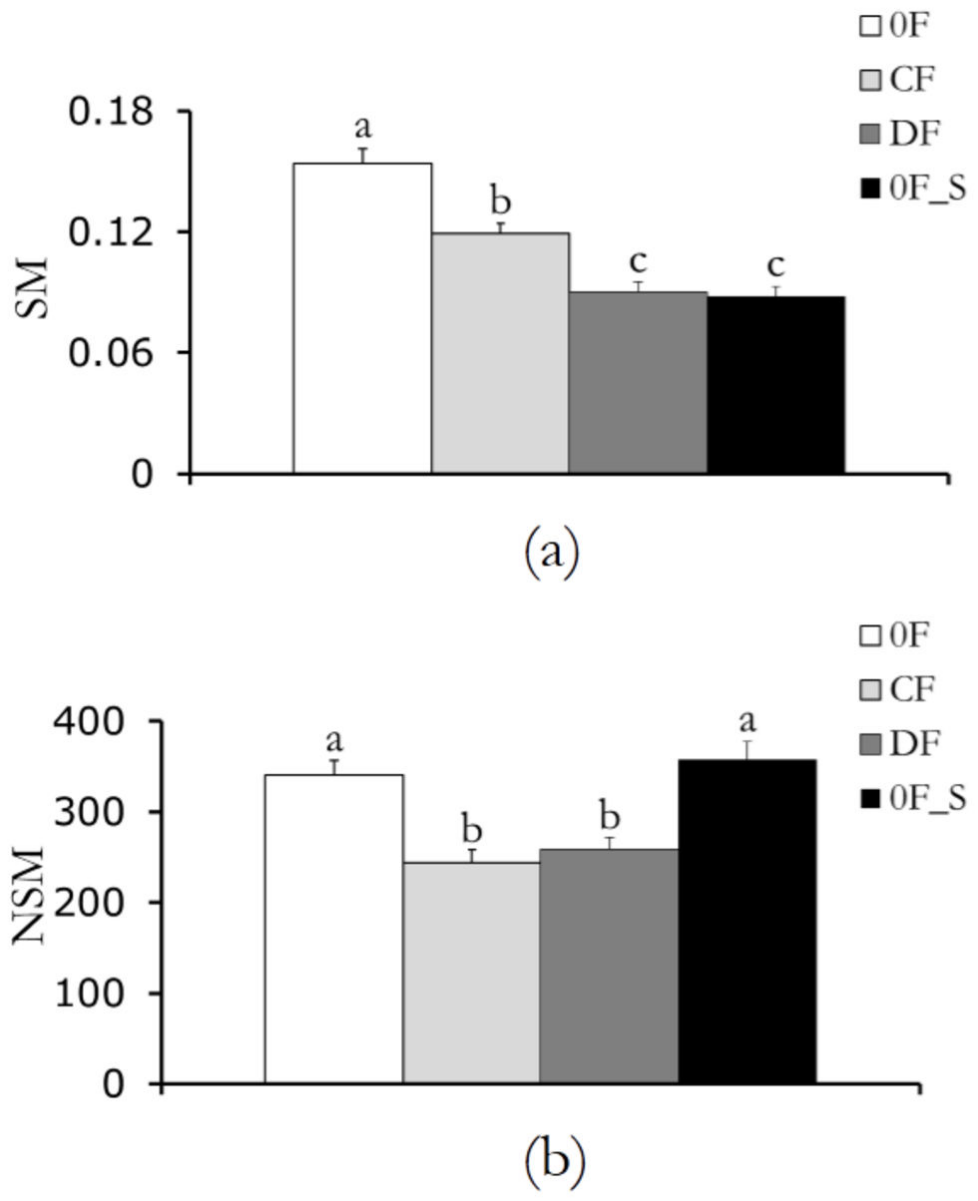
Movement smoothness: (a) mean of the speed metric (SM) for the four conditions with error bars representing standard error and (b) mean of the number of submovements (NSM) for the four conditions, with error bars representing standard error. Bars with different letters are statistically different from post-hoc comparisons.

### Force feedback and scientific learning regulate movement accuracy (Figure 4)

Our results also confirm the expectation that the accuracy of participants’ movements is influenced by the experimental condition (for D F3,48=9.519, p<0.0001 and NPL F3,48=54.909, p<0.0001). Specifically, we find that the deviation D is minimized for CF, where a converging force feedback is offered to the subjects (p<0.0005 in post-hoc tests comparing CF to any other condition). The analysis of the normalized path length NPL indicates that the diverging force feedback in condition DF maximizes the length of the track paths taken by the subjects (p<0.0001 in post-hoc tests comparing DF to any other condition). In addition, we find a significant difference when comparing NPL for 0F and CF, suggesting that the presence of a converging force feedback increases the actual path length taken by subjects (p=0.0395 in post-hoc tests). 

**Figure 4:**
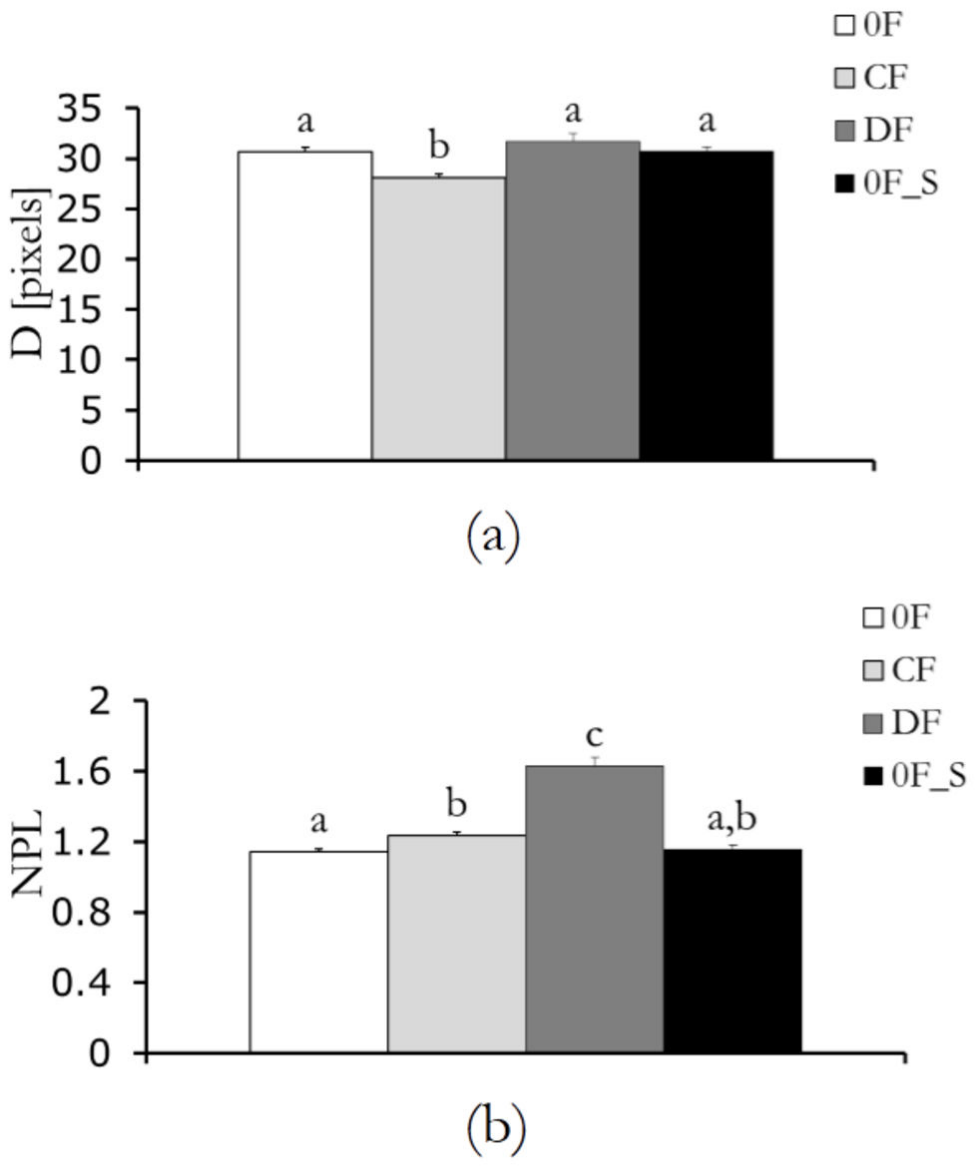
Movement accuracy: (a) mean of the deviation (D) for the four conditions with error bars representing standard error and (b) mean of the normalized path length (NPL) for the four conditions, with error bars representing standard error. Bars with different letters are statistically different from post-hoc comparisons.

### Force feedback and scientific learning regulate tracking rapidity (Figure 5)

Our results indicate that the task duration T is influenced by the experimental condition (F3,48=2.713, p=0.0462). Post-hoc comparisons reveal that the presence of a converging feedback in CF reduces the task duration as compared to both DF and 0F_S, in which the participants are offered a diverging force feedback or are exposed to the scientific content (p=0.0400 in post-hoc test comparing CF and DF and p=0.146 comparing CF and 0F_S). 

**Figure 5:**
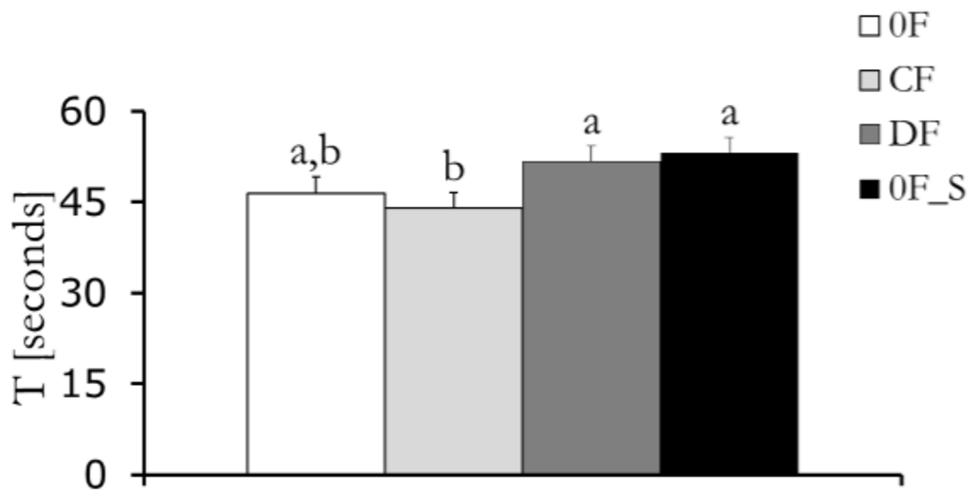
Mean of the task duration (T) for the four conditions with error bars representing standard error.

### Scientific content is associated with increased participants’ satisfaction

A comparison between the learning-based condition 0F_S and the control condition 0F demonstrate that the satisfaction from performing the task was higher in the learning-based task (4.08 vs. 3.79). A paired-samples t-test show that the difference is significant (p=0.0301).

### Scientific learning increases with satisfaction (Figure 6)

Out of 48 subjects, 28 participants showed a nonzero difference; in other words, for 28 users there was a difference between the satisfaction levels in the two tasks. For these 28 users, a positive, yet not significant, correlation of 0.27 (p = 0.17) was found between satisfaction difference and learning. 

**Figure 6:**
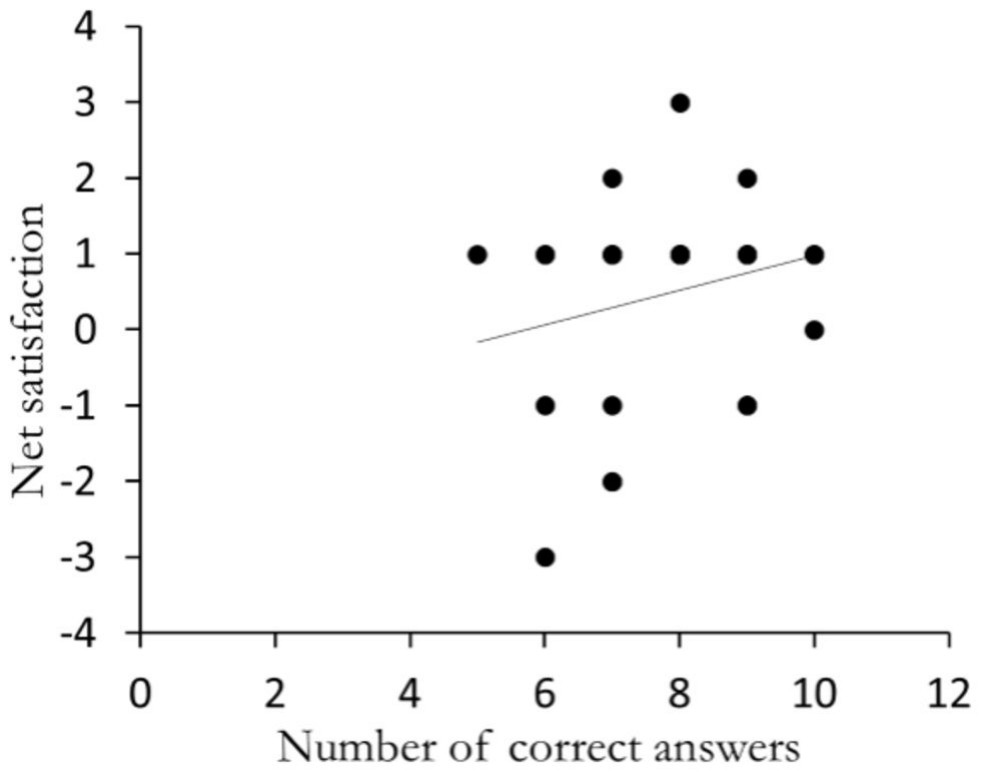
Correlation between the number of correct answers and the net satisfaction. Solid line is a linear regression.

## Discussion

In the present study, we show that healthy adult subjects respond differentially to the administration of force feedback and scientific content in a virtual environment, where they interact with a low-cost haptic device to control the movement of a cursor on a predefined trajectory. The virtual environment is designed based on a map of a local zoo, which offers several opportunities for delivering scientific content to the subjects during the task execution. The proposed low-cost system enables the delivery of RMT and performance assessment through a suite of objective indices, based on variables validated in previous studies [41-47] that quantify the subject’s dexterity in planning and generating multijoint visuomotor tasks. 

### Force feedback regulates the smoothness, accuracy, and duration of the subject’s movement

The analysis of movement smoothness (using SM and NSM) indicates that when converging or diverging force fields are applied, the range of variations of the hand speed increases with respect to the control condition 0F, as evidenced by a reduction of SM and NSM (without an increase in the task duration). We propose that this effect reflects the subjects’ effort to counter the real-time force feedback by exerting a continuous and fine regulation of the end-effector. These results are consistent with findings from [49], where an ad-hoc robotic platform is utilized to test the hypothesis as to whether the smoothness of the targeting movements is influenced by an attractive force field in hemiparetic patients. In addition to confirming such effect in healthy subjects through a low-cost haptic device, we demonstrate that a further enhancement is possible through the use of a diverging force field. Specifically, while the number of submovements does not vary with the type of force feedback, a significant decrease in the speed metric is observed in the presence of a diverging force field. This posits the need for future work on assessing the feasibility of tailoring functional recovery through the integration of varying or adaptive force feedback strategies. 

### Force feedback from low-cost haptic devices can be used for upper limb rehabilitation

Motor program reorganization induced by converging and diverging force fields is likely related to a trial and error process, in which subjects selectively activate the muscular-skeletal system in response to the external perturbations. Thus, the delivery of an external force causes fine and continuous on-line adjustments of the hand positions on the basis of the discrepancy between the target and the cursor locations. These voluntary compensatory activities are evident in our results concerning the normalized path length and the trial duration, which demonstrate variations in the subjects’ response due to force feedback. In future studies, these performance evaluation metrics will be used to update the robot control in real-time, during the execution of a motor exercise for rehabilitation treatments of patients with paretic upper limbs. 

### The inclusion of science learning in the exercise procedure increases participants’ interest

Another important finding of our study concerns the utility of combining learning in the tele-rehabilitation process. While preliminary, our results suggest that using scientific content increases subjects’ satisfaction. This is a relevant aspect in neuro-rehabilitation research, since a major problem is patients’ boredom resulting from performing repetitive tasks [50]. Increasing the level of interest and including learning in the process can achieve two goals: (i) increasing the time spent on performing rehabilitation tasks and (ii) leveraging these tasks towards learning in a new context. In future studies, with larger samples of subjects, it will be possible to analyze more thoroughly: (i) the direct relationship between subjects’ engagement with scientific content and their rehabilitation performance and (ii) the effect of the interaction with different content types on subjects’ satisfaction and performance. Further, these future studies will evaluate the intertwined effects of engagement in scientific content and force feedback on rehabilitation outcomes. With respect to sensory-motor performance, the administration of scientific content is only responsible for a significant decrease in the speed metric with respect to the control condition. This difference should be ascribed to the segmentation of the trajectory in 0F_S, which induces a relevant decrease in the average speed. Specifically, the reaction time of the subjects from the closing of the scientific content window to resuming the task is responsible for a few stagnations in the cursor position.

In conclusion, our findings offer evidence for the utility of low-cost haptic devices in evaluating and training the upper limb by tracking predefined trajectories in a 2D virtual environment. Specifically, our results support the hypotheses that force feedback regulates salient indices of sensory-motor performance and that the inclusion of science learning fosters interest in participation. Beyond these theoretical contributions, this work addresses a wide spectrum of methodological aspects that can be leveraged in the design of neuro-rehabilitation hypothesis-driven studies based on the Novint Falcon, as well as alternative platforms for remote tele-rehabilitation by home users. Methodological elements that are advanced by this work include: (i) real-time feedback as a function of end-effector motion; (ii) ad-hoc administration of scientific content; (iii) establishment of a toolbox of sensory-motor performance indices; and (iv) design of content-specific survey instruments to evaluate engagement and learning. Future studies will be focused on implications of these results in rehabilitation contexts, whereby a similar setting will be used to treat upper limb paresis and stimulate patients’ participation through the presentation of relevant scientific content. As part of this work, we will also explore the feasibility of optimizing the administration of scientific information, including their content and distribution in the 2D virtual environment, to enhance participation.
